# Preclinical support for the therapeutic potential of zolmitriptan as a treatment for cocaine use disorders

**DOI:** 10.1038/s41398-020-00956-6

**Published:** 2020-08-03

**Authors:** Raul Garcia, Tien Le, Samantha N. Scott, Delaram Charmchi, Jamie M.L. Sprout, Nathan S. Pentkowski, Janet L. Neisewander

**Affiliations:** 1 School of Life Sciences, Arizona State University, Tempe, AZ USA; 2 School of Engineering of Matter, Transport, and Energy, Arizona State University, Tempe, AZ USA; 3 Present Address: Department of Psychology, University of New Mexico, Albuquerque, NM USA

**Keywords:** Pharmacology, Neuroscience

## Abstract

Serotonin 1B receptor (5-HT_1B_R) agonists enhance cocaine intake in rats during daily self-administration but attenuate cocaine intake after prolonged abstinence. Here we investigated whether the less selective but clinically available 5-HT_1D/1B_R agonist, zolmitriptan, produces similar effects. Male and free-cycling female Sprague-Dawley rats were trained to lever press for cocaine (0.75 mg/kg, i.v.) or sucrose (45 mg pellet) reinforcement until performance rates stabilized. Rats then received zolmitriptan (3.0, 5.6, and 10 mg/kg, s.c.) prior to testing for its effects on response and reinforcement rates. Under cocaine testing conditions, rats had access to the training dose for the first hour followed by a lower cocaine dose (0.075 mg/kg, i.v.) for the second hour. Zolmitriptan decreased cocaine intake at both cocaine doses and in both sexes even without a period of abstinence and without altering sucrose intake. A separate group of rats underwent identical training procedures and were tested for effects of the selective 5-HT_1B_ and 5-HT_1D_ receptor antagonists, SB224289 (3.2, 5.6, and 10 mg/kg, s.c.) and BRL15572 (0.3, 1.0, and 3.0 mg/kg, i.p.), respectively, alone or in combination with zolmitriptan (5.6 mg/kg, s.c.) under identical cocaine testing procedures as above. The zolmitriptan-induced decrease in cocaine intake was reversed by SB224289 and to a lesser extent by BRL15572, suggesting that the effects of zolmitriptan involve both 5-HT_1B_ and 5-HT_1D_ receptors. Neither zolmitriptan, SB224289, or BRL15572 altered locomotor activity at the doses effective for modulating cocaine intake. These findings suggest that zolmitriptan has potential for repurposing as a treatment for cocaine use disorders.

## Introduction

Despite recent increases in cocaine use and deaths related to cocaine overdose^1–3^, no effective treatment for cocaine use disorders (CUDs) exists. We and others have suggested that serotonin 1B receptors (5-HT_1B_Rs) may be suitable targets for CUDs medication development^4–7^. Initial studies using 5-HT_1B_R agonists found enhancement of cocaine self-administration and cocaine conditioned place preference^8–11^ but attenuation of amphetamine self-administration^12,13^. We later found that a selective 5-HT_1B_R agonist, CP94253, or over-expression of 5-HT_1B_Rs modulate cocaine self-administration differently depending on the stage of the drug-taking cycle. Both manipulations increase cocaine intake on a fixed ratio 5 schedule of reinforcement during daily maintenance of self-administration but decrease cocaine intake following a period of forced abstinence^14–16^. Similarly, CP94253 dose-dependently decreases cocaine-seeking behavior and cocaine intake on a progressive ratio reinforcement schedule after a period of forced abstinence^14,16^. These inhibitory effects on cocaine reinforcement/motivation suggest that 5-HT_1B_R agonists may have therapeutic potential for preventing relapse after a period of abstinence.

Tryptamine-based 5-HT_1B_R agonists, referred to as triptans, are used clinically for treatment of migraine headaches^17^. The migraine medication zolmitriptan is a nonselective 5-HT_1D/1B_R agonist with slightly higher affinity for 5-HT_1D_Rs over 5-HT_1B_Rs (Ki = 0.63 vs. 5.01 nM, respectively)^18^. Zolmitriptan readily crosses the blood-brain barrier^17,19–21^, and is effective in relieving migraine headaches following long-term use in adolescent and adult male and female patients^21–23^. These qualities make zolmitriptan a suitable candidate for preclinical investigation of its potential as a treatment for CUDs.

In the present study, we examined the dose-effect function of zolmitriptan on cocaine self-administration in male and female rats during maintenance of daily 2-h access to cocaine. Based on our previous findings with 5-HT_1B_R agonists and cocaine, we hypothesized that zolmitriptan would increase cocaine intake during maintenance of self-administration and decrease cocaine intake following prolonged abstinence in male rats and that these effects would also extend to female rats. However, in contrast to the selective 5-HT_1B_R agonists tested in the past, zolmitriptan reduced cocaine intake during maintenance of self-administration and this effect occurred regardless of sex. We then examined if the zolmitriptan-induced effects were 5-HT_1B_R- and/or 5-HT_1D_R-mediated using selective antagonists. Zolmitriptan effects were blocked by a selective 5-HT_1B_R antagonist and to some extent by a 5-HT_1D_R antagonist. As a control for general disruption of operant/consummatory behaviors, we also examined zolmitriptan effects on surcrose reinforcement and spontaneous locomotion. Doses of zolmitriptan that were effective at reducing cocaine intake failed to alter sucrose intake or locomotion.

## Material and methods

### Subjects

Male and free-cycling female Sprague Dawley rats (~postnatal day 60 at the start of the experiment; Charles River, CA, USA) weighing 200–300 g were single-housed in separate climate-controlled environments on a 14:10 reverse light/dark cycle (lights off at 6 am). Rats were handled for 5–6 days prior to beginning any procedure and were given *ad libitum* access to food and water except for initial cocaine and sucrose self-administration training when they were food-restricted to 85% of their *ad libitum* weights to facilitate acquisition of operant responses. Studies have shown that ovarian hormones influence cocaine-related behaviors^24–26^. However, our lab has found no estrous cycle differences in cocaine self-administration in female rats undergoing chronic cocaine self-administration similar to that used here, nor have we found any difference in the effects of a selective 5-HT_1B_R agonist on cocaine self-administration in female rats across estrous cycle phases^27^. Therefore, we did not monitor the estrous cycle in this study. Sample size was determined based on similar procedures from previously published studies^14,28^. The experiments proceeded in accordance with a protocol approved by the Arizona State University Institutional Animal Care and Use Committee.

### Drugs

Cocaine hydrochloride (RTI International, NC, USA) was dissolved in saline and filtered with Acrodisc syringe filters (PALL Corporation, NY, USA). Zolmitriptan, SB224289, and BRL15572 (Tocris Bioscience, MN, USA) were dissolved in dimethyl sulfoxide (DMSO) and diluted with saline to a final concentration of 10% DMSO. Additionally, high doses of SB224289 were sonicated for ~2 mins with gentle warming. SB224289 and BRL15572 were selected for their high affinity and selectivity as antagonists for 5-HT_1B_Rs and 5-HT_1D_Rs, respectively^29,30^. All drug injections, except for self-administered cocaine, were prepared fresh daily and injected at a volume of 1 ml/kg body weight. Vehicle refers to the respective solvent.

### Surgery

Rats for the cocaine self-administration experiments underwent surgery for implanting a chronic indwelling catheter into the jugular vein as detailed in Garcia et al.^28^. Rats had 5–6 days of recovery before commencing cocaine self-administration training. During the recovery period, catheters were flushed with cefazolin (10 mg/0.1 ml, i.v.; WG Critical Care, NJ, USA) mixed with heparin/saline (7 u/0.1 ml; APP Pharmaceuticals, IL, USA). After recovery, catheters were flushed only with heparin/saline. Catheter patency was tested periodically with the short-acting barbiturate methohexital sodium (0.835 mg/0.05 ml infusion; Jones Pharma Inc., MO, USA) at a dose that produces brief loss of muscle tone only when administered i.v.

### Apparatus

The operant conditioning chambers (Med Associates, VT, USA) contained an active and inactive lever, a cue light, and a tone generator as previously described in Pentkowski et al.^16^. The chambers contained either an infusion pump (Med Associates) connected to a liquid swivel (Instech, PA, USA) and attached to a polyethylene tubing sheltered within a metal leash (PlasticsOne, VA, USA) or contained pellet dispensers (Med Associates). All operant conditioning chambers were housed within sound attenuating boxes that contained a ventilation fan. Male and female rats underwent self-administration in different rooms to avoid potential confounding influences of sex on behavior.

### Self-administration training

Sessions commenced at approximately the same time of day, 6–7 days/week during the rats’ dark cycle. Rats were trained to lever press on a fixed ratio (FR) 1 schedule of cocaine reinforcement (0.75 mg/kg/0.1 ml infusion, i.v.) or sucrose reinforcement (45 mg pellet, Bio-Serv). Each session began by extending both levers and all other self-administration training procedures were identical to Pentkowski et al.^14^. Briefly, upon schedule completion, light and tone cues were activated and 1 s later the cocaine infusion was delivered over 6 s or a sucrose pellet was dispensed. Cues were then terminated and a house light was illuminated to signal a 20-s time out period during which no reinforcement was available. Once rats met the training criterion of >10 reinforcers by the end of each session for two consecutive sessions they progressed to an FR5 reinforcement schedule and after meeting the training criterion again, they were gradually placed on *ad libitum* feeding. Testing procedures began once rats met a stability criterion of ≤15% variance in total number of reinforcers obtained on the FR5 schedule across three consecutive sessions without food restriction. Investigator performing treatment injections on test sessions remained the same and investigators assisting with behavioral testing procedures were single blinded to treatment conditions.

### Zolmitriptan effects on cocaine self-administration

Upon meeting the stability criterion described above on the FR5 schedule of cocaine reinforcement, rats were tested repeatedly for effects of zolmitriptan using a within-subjects design. All doses were chosen based on previous research indicating the effectiveness and safety profile of zolmitriptan in rodents and human subjects^18,31^. Fifteen min prior to test sessions, rats received either 3.0, 5.6, or 10 mg/kg, s.c. zolmitriptan in descending order across tests. Rats also received a vehicle treatment test in between each of the three zolmitriptan tests, with order of vehicle and zolmitriptan tests counterbalanced. Rats tested under the two different orders (i.e., zolmitriptan vs. vehicle first) were matched as closely as possible for the number of infusions obtained prior to the test (Table 1). During test sessions, rats had access to the training dose (0.75 mg/kg, i.v.) for the first hour and then to a low dose of cocaine (0.075 mg/kg, i.v.) for the second hour. These cocaine doses were selected based on previous experiments in our lab demonstrating that they fall on the descending and ascending limbs, respectively, of the self-administration dose-effect function^14^. Treatment-induced changes with these doses can be interpreted as changes in the reinforcing value of cocaine^32^. For example, a leftward shift of the dose-response curve is indicated by an increase in cocaine intake at the low dose and a decrease in intake at the high dose^14^, and is interpreted as an enhancement in cocaine reinforcing value. Similarly, a downward shift of the dose-response curve is indicated by a decrease in cocaine intake at both doses and is interpreted as an attenuation in cocaine reinforcing value. The order of cocaine dose was chosen to avoid difficulties stabilizing performance when the low dose is presented first. Between test sessions, rats received additional 2-h sessions with access to the training dose until the stability criteria were re-established.

**Table 1:** Baseline values for number of cocaine (0.75 mg/kg, i.v.) infusions/2 h.

Treatment assignment	^1^Infusion baselines (±SEM)			
Dose (mg/kg)	Males	*n*	Females	*n*
Zol 0	23.87 (±1.28)	14	23.57 (±1.62)	14
Zol 3.0	23.48 (±0.95)	14	26.19 (±2.38)	14
Zol 5.6	22.76 (±0.99)	14	25.10 (±2.15)	14
Zol 10	23.02 (±1.52)	14	23.71 (±2.03)	14
SB 3.2	27.56 (±1.67)	13	30.33 (±3.18)	10
SB 5.6	28.57 (±2.40)	14	27.02 (±1.33)	10
SB 10	28.10 (±2.11)	13	29.70 (±3.28)	10
SB 3.2 + Zol 5.6	26.58 (±2.44)	13	29.85 (±1.51)	10
SB 5.6 + Zol 5.6	27.73 (±2.63)	13	28.05 (±1.86)	10
SB 10 + Zol 5.6	27.57 (±1.92)	14	27.82 (±2.12)	10
BRL 0.3	26.90 (±1.73)	10	22.50 (±1.58)	8
BRL 1.0	28.43 (±2.25)	14	27.42 (±1.54)	11
BRL 3.0	27.85 (±1.96)	14	27.47 (±2.95)	12
BRL 0.3 + Zol 5.6	26.72 (±1.64)	10	22.10 (±1.68)	8
BRL 1.0 + Zol 5.6	27.92 (±1.92)	12	27.49 (±2.96)	13
BRL 3.0 + Zol 5.6	28.07 (±2.33)	12	26.44 (±2.20)	13

^1^Average of the three consecutive baseline sessions prior to each treatment test; SB = SB224289; BRL = BRL15572; Zol = Zolmitriptan.

### Zolmitriptan effects on sucrose reinforcement

Experimentally naïve male (*n* = 10) and free-cycling female (*n* = 14) rats underwent the same training and testing procedures as in experiment 1 except that sucrose (45 mg) was the reinforcer.

### Effects of antagonists and zolmitriptan on cocaine reinforcement

Experimentally naive rats underwent the same training procedures as described in experiment 1. Additionally, a subset of rats from experiment 1 were used. Due to attrition from catheter failure, the number of rats tested varied and is reported in Table 1. After meeting the stability criteria, rats were randomly assigned to receive vehicle, SB224289 (3.2, 5.6, or 10 mg/kg, s.c.), or BRL15572 (0.3, 1, or 3 mg/kg, i.p.) alone or in combination with zolmitriptan (5.6 mg/kg, s.c.). Prior to each test, rats received two injections 15 min apart, first of their assigned antagonist dose followed by zolmitriptan or vehicle. Testing began 15 min after the latter injection. All other testing procedures were identical to the procedures described above.

### Effects of 5-HT_1B_R and 5-HT_1D_R drugs and locomotion

A subset of rats from experiment 3 were placed into abstinence for a minimum of 10 days following the last test for antagonist effects on cocaine self-administration. Rats were tested for effects of vehicle, zolmitriptan (5.6 mg/kg, s.c.) or SB224289 (10 mg/kg, s.c) on spontaneous locomotion using a within-subjects design. After completing these tests, rats underwent 5 rest days in their home cage and then began tests for the effects of vehicle or BRL15572 (0.3, 1.0, & 3.0 mg/kg, i.p.) on spontaneous locomotion using a within-subjects design. SB224289 and BRL15572 were injected 30 min prior to testing. In addition, the SB224289 experiment rats received an injection 15 min later of either vehicle or zolmitriptan (5.6 mg/kg, s.c.). The test began by placing the rats into testing chambers (45.72 × 25.4 × 20.32 cm) that had a camera mounted above to record horizontal movement (Topscan, VA, USA). Test sessions lasted 60 min. The SB224289 dose chosen was effective in reversing the attenuating effects of zolmitriptan on self-administration. Limited evidence exists regarding 5-HT_1D_R antagonists and locomotor activity and therefore multiple doses were examined. Rats had two-three rest days in between test sessions where they remained in their home cage. All treatments were randomly assigned with order counterbalanced.

### Statistical analysis

Statistical analyses were conducted with IBM® SPSS Statistics v. 25 without the use of condition blinding. Self-administration data, including reinforcers obtained, intake (mg/kg, i.v.), active and inactive lever responses, as well as total distance traveled for locomotion were analyzed by ANOVA with repeated measures and corrected for sphericity using the Greenhouse-Geisser method when applicable. Due to attrition, we had missing data that precluded using a within-subjects repeated measure analysis for antagonist dose and this variable was treated as a between-subjects variable. Analyses were conducted using two-tailed tests and all sources of significant effects were further analyzed by Tukey’s post-hoc tests.

## Results

All descriptive statistics are reported as the mean ± SEM. Significance threshold (α) was set at *p* < 0.05 for all comparisons.

### Acquisition of cocaine self-administration

There were sex differences in the number of cocaine infusions obtained during acquisition [F(1,52) = 8.224, *p* < 0.05], which were largely due to males having higher infusion rates early during training (data not shown). The average reinforcement rate/2 h in males and females across sessions was 21.20 ± 0.57 and 16.82 ± 0.58, respectively (*p* < 0.05). During the final three days of training when reinforcement rates were stable, this sex difference was no longer evident, and all baseline reinforcement rates prior to each test with zolmitriptan showed no effects of sex, day, nor interaction (Table 1).

### Zolmitriptan effects on cocaine reinforcement

All omnibus analyses indicated no main effect or interactions with sex, and therefore the data were collapsed across males and females for subsequent analyses. Because there were no effects of vehicle pretreatment across each of the three vehicle tests occurring between each of the zolmitriptan dose pretreatments on any of the measures, the average of all three vehicle tests was used in further analyses to achieve the most representative value and to simplify presentation.

**Fig. 1:**
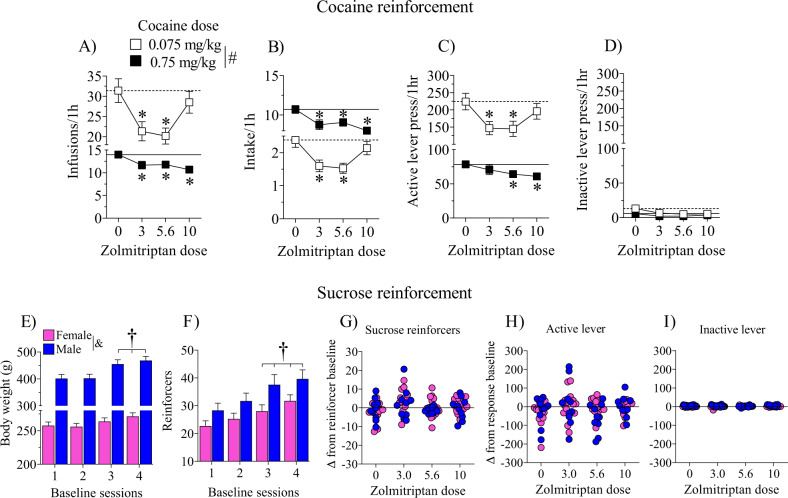
Effects of the 5-HT1D/1B receptor agonist, zolmitriptan, on behaviors under cocaine and sucrose reinforcement. There were no main effects nor interactions with sex for any of the operant behavioral measures and therefore these data are collapsed across sex. Mean (±SEM) is shown for cocaine infusions (**a**), intake (mg/kg) (**b**), active (**c**) and inactive (**d**) lever responses during 2-h sessions where the training dose (0.75 mg/kg, i.v.) of cocaine was available for 1h followed by a low dose (0.075mg/kg, i.v.) of cocaine available for 1h. Horizontal lines correspond to the mean of vehicle pretreatment under the low (dashed line) and training (solid line) cocaine doses. Rats tested for sucrose reinforcement showed sex differences in body weight (**e**) and reinforcement baselines (**f**). Therefore, the effects of zolmitriptan are expressed as a difference from each respective baseline for sucrose reinforcers (**g**), and active (**h**) and inactive (**i**) lever responses during 30-min sessions. All rats (*n* = 28/dose for cocaine and *n* = 24/dose for sucrose) were pretreated 15 min prior to the start of the test sessions with either vehicle or zolmitriptan (3.0–10 mg/kg, s.c.). Asterisks (*) represent a difference from vehicle, pound sign (#) represents a difference between low and high cocaine dose, dagger (†) represents difference from first baseline session, and ampersand (&) represents difference between male and female rats (*p* < 0.05).

Zolmitriptan decreased the number of cocaine infusions obtained, intake (mg/kg, i.v.), and active lever responses. Analyses revealed main effects of cocaine dose [*F*(1,27) = 81.61, 617.65 and 69.45, respectively, *p’s* < 0.05], zolmitriptan dose [*F*(3,81) = 7.46, 7.76, and 5.91, respectively, *p’s* < 0.05], and cocaine dose by zolmitriptan dose interactions [*F*(3,81) = 6.33, 6.31, and 4.84, respectively, *p’s* < 0.05]. Rats received a higher number of cocaine infusions and pressed the active lever more while the low cocaine dose was available, but their cocaine intake was higher when the high cocaine dose was available as expected (*p’s* < 0.05). More importantly, zolmitriptan at 3.0 and 5.6 mg/kg decreased cocaine infusions (Fig. 1a), intake (Fig. 1b), and active lever responses (Fig. 1c) when 0.075 mg/kg cocaine was available, and at all doses of zolmitriptan (3.0–10 mg/kg, s.c.) when 0.75 mg/kg cocaine was available (Fig. 1a–c; *p’s* < 0.05). Surprisingly, zolmitriptan at 10 mg/kg did not attenuate cocaine intake when the low dose of cocaine was available. Neither cocaine nor zolmitriptan dose influenced inactive lever responses (Fig. 1d).

### Zolmitriptan effects on sucrose reinforcement

Although overall operant measures showed no sex differences during initial training (data not shown), sex differences emerged during the final three days of training [*t(*4) = 8.88, *p* < 0.05], evident as higher mean reinforcers obtained/30 min in males (28.33 ± 0.60) than females (22.71 ± 0.18). This sex difference is expected given the differences in body weight because males gained more weight than females as testing progressed (baseline weights by sex interaction [*F*(3,66) = 17.68, *p* < 0.05] (Fig. 1e). Similarly, baseline sucrose reinforcement rates increased across time [*F*(3,66) = 25.94, *p* < 0.05] and were higher in males than females [*F*(1,22) = 5.00, *p* < 0.05] (Fig. 1f). To correct for these differences, subsequent analyses were performed on the change from baseline. Analyses indicated no sex or zolmitriptan main effects nor interactions for sucrose reinforcers obtained (Fig. 1g), or active (Fig. 1h) and inactive (Fig. 1i) lever responses.

### Effects of antagonists and zolmitriptan on cocaine reinforcement

**Fig. 2:**
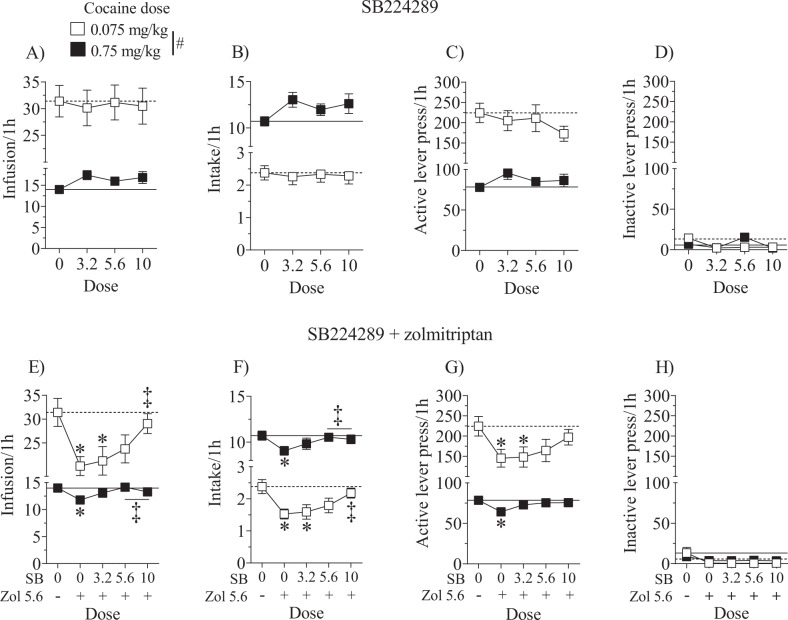
Effects of the 5-HT1B receptor antagonist, SB224289, alone or in combination with zolmitriptan. There were no main effects nor interactions with sex and therefore the data are collapsed across sex. Data are expressed as the mean (±SEM) for cocaine infusions (**a**, **e**), intake (**b**, **f**), and active (**c**, **g**) and inactive (**d**, **h**) lever responses during test sessions where the training dose of cocaine (0.7 5mg/kg, i.v.) was available for 1h followed by a low dose of cocaine (0.075mg/kg,i.v.) available for 1h. Horizontal lines correspond to the mean of vehicle pretreatment under the low (dashed line) and training (solid line) cocaine doses tested. Rats (*n* = 20–28/dose) were pretreated 30min prior to the start of the test sessions with SB224289 (3.2–10 mg/kg, s.c.) followed by either vehicle or zolmitriptan (5.6 mg/kg, s.c.) 15 min later. Pound sign (#) represents a difference between low and high cocaine dose, asterisks (*) represent adifference from vehicle, and double dagger (‡) represents difference from zolmitriptan alone (*p* < 0.05).

The effects of the selective 5-HT_1B_R and 5-HT_1D_R antagonists on cocaine self-administration were assessed both alone and in combination with zolmitriptan. Initial analyses of baseline reinforcement rates and test day measures showed no effects of sex (Table 1) and therefore data are collapsed across sex for subsequent analyses. Analyses of SB224289 effects when given alone revealed only main effects of cocaine dose for infusions, intake (mg/kg, i.v.), and active lever responses [*F*(1,94) = 106.07, 849.12 and 100.61, respectively, *p’s* < 0.05]. While rats received a higher number of cocaine infusions (Fig. 2a) and pressed the active lever more (Fig. 2c) during availability of the low cocaine dose, they consumed more cocaine when the high cocaine dose was available as expected (Fig. 2b; *p* < 0.05). There were no effects on inactive lever responses (Fig. 2d). Overall, treatment with SB224289 alone did not alter cocaine self-administration.

SB224289 reversed the zolmitriptan-induced attenuation of cocaine self-administration behaviors. Analyses of cocaine infusions, intake (mg/kg, i.v.), and active lever responses all revealed main effects of cocaine dose [*F*(1,116) = 114.46, 1305.14, and 102.95, respectively, *p’s* < 0.05], SB224289 dose [*F*(4,116) = 3.58, 2.58, and 2.46, respectively, *p’s* < 0.05] and cocaine dose by SB224289 dose interactions [*F*(4,116) = 3.46, 8.60, and 2.99, respectively, *p’s* < 0.05]. While rats received a higher number of cocaine infusions (Fig. 2e) and pressed the active lever more (Fig. 2g) during availability of the low cocaine dose, they consumed more cocaine when the high cocaine dose was available as expected (Fig. 2f; *p* < 0.05). Zolmitriptan (5.6 mg/kg, s.c.) alone attenuated cocaine infusions (Fig. 2e), intake (Fig. 2f), and active lever responses (Fig. 2g, *p’s* < 0.05) as expected. Importantly, SB224289 dose-dependently reversed the attenuating effects of zolmitriptan, with no differences from vehicle pretreatment noted at the two highest doses of SB224289 across all of these measures. Furthermore, the highest doses appeared to completely reverse the zolmitriptan-induced reduction of infusions and intake as these doses were different from zolmitriptan alone (*p’s* < 0.05). There were no effects on inactive lever responses (Fig. 2h). Collectively, these results indicate that zolmitriptan decreases cocaine intake in a 5-HT_1B_R-mediated fashion.

**Fig. 3:**
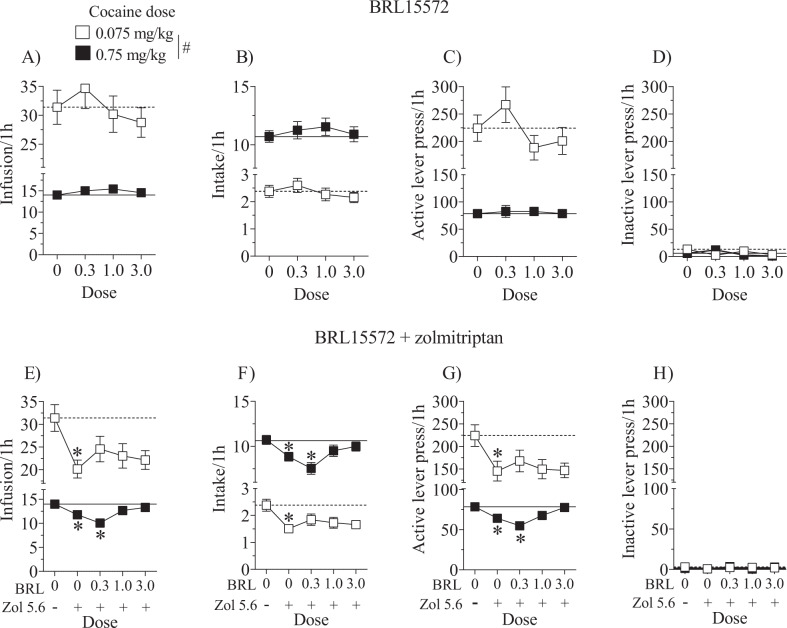
Effects of the 5-HT1D receptor antagonist, BRL15572, alone or in combination with zolmitriptan. There were no interaction effects with sex and pretreatment and therefore the data are collapsed across sex. Data are expressed as the mean (±SEM) for cocaine infusions (**a**, **e**), intake (**b**, **f**), and active (**c**, **g**) and inactive (**d**, **h**) lever responses during test sessions where the training dose of cocaine (0.75 mg/kg, i.v.) was available for 1h followed by a low dose of cocaine (0.075 mg/kg, i.v.) available for 1h. Horizontal lines correspond to the mean of vehicle pretreatment under the low (dashed line) and training (solid line) cocaine doses tested. Rats (*n* = 18–28/dose) were pretreated 30min prior to the start of the test sessions with BRL15572 (0.3–3 mg/kg, i.p.) followed by either vehicle or zolmitriptan (5.6 mg/kg, s.c.) 15min later. Pound sign (#) represents a difference between low and high cocaine dose, and asterisks (*)represent a difference from vehicle (*p* < 0.05).

The selective 5-HT_1D_R antagonist, BRL15572 given alone had no effect on cocaine infusions, intake, or active lever responses. There were only main effects of cocaine dose for these respective measures [*F*(1,88) = 144.69, 919.75, and 138.23, *p’s* < 0.05; Fig. 3a–c], replicating the expected effects observed in the previous experiments. There were no effects or interactions in the analysis of inactive lever responses (Fig. 3d). These results indicate that treatment with BRL15572 alone did not alter cocaine self-administration.

BRL15572 partially reversed the zolmitriptan-induced attenuation of behavioral measures. For cocaine infusions and active lever responses, unlike all other analyses in this study, there was a main effect of sex [*F*(1,111) = 8.09 and 5.84, respectively, *p’s* < 0.05] and a cocaine dose by sex interaction [*F*(1,111) = 7.51 and 6.59, respectively, *p’s* < 0.05] where female rats took fewer infusions and responded less on the active lever than male rats only when the low dose of cocaine was available (Figure S1, *p* < 0.05). Additionally, for cocaine infusions, intake, and active lever responses there were main effects of cocaine dose [*F*(1,111) = 134.86, 1069.95, and 114.97, respectively, *p’s* < 0.05], BRL15572 dose [*F*(4,111) = 4.22, 4.58, and 3.00, respectively, *p’s* < 0.05], and cocaine dose by BRL15572 dose interactions [*F*(4,111) = 3.32, 3.79, and 2.52, respectively, *p’s* < 0.05]. Zolmitriptan (5.6 mg/kg, s.c.) alone significantly decreased cocaine infusions (Fig. 3e), intake (Fig. 3f), and active lever responses as expected (Fig. 3g; *p* < 0.05) and BRL15572 attenuated this effect except at the lowest dose when the high dose of cocaine was available. In summary, BRL15572 was not as efficacious at reversing the effects of zolmitriptan when the low dose of cocaine was available, in part, because these effects were less pronounced in female rats at the low cocaine dose. However, BRL15572 at 1.0 and 3.0 mg/kg (i.p.) reversed the attenuating effects of zolmitriptan when the high cocaine dose was available suggesting that zolmitriptan’s effects also involve 5-HT_1D_ receptors. There were no effects on inactive lever responses (Fig. 3h).

### Effects of 5-HT_1B_R and 5-HT_1D_R drugs and locomotion

**Fig. 4:**
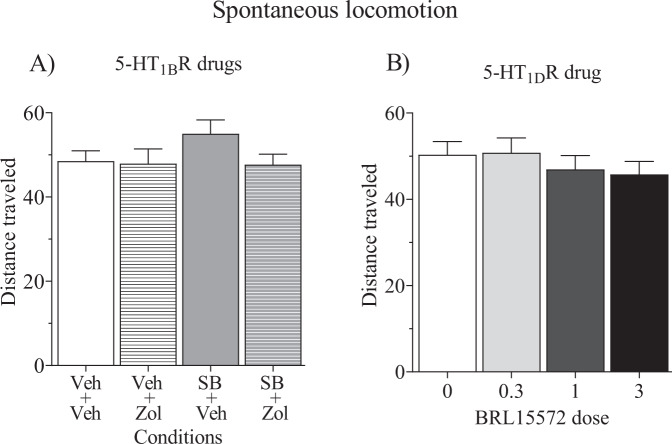
Effects of test drugs on spontaneous locomotion. Locomotor activity expressed as the mean (±SEM) is shown for total distance traveled in meters (m) across a 1-h session. Rats were pretreated 30min prior to the start of the session with either vehicle, SB224289 (10 mg/kg, s.c.) or BRL15572 (0–3.0 mg/kg, i.p.). Rats in the SB224289 experiment received a second pretreatment of either vehicle or zolmitriptan (5.6 mg/kg, s.c.) 15min later. There were no main effects nor interactions; data are collapsed across male (*n* = 6) and female (*n* = 7) rats.

Drug pretreatment effects on locomotor activity revealed no sex differences and therefore locomotor activity data was collapsed across male and female rats. Analyses of total distance traveled revealed no effects of 5-HT_1B_R drugs (Fig. 4a) or 5-HT_1D_R drugs (Fig. 4b) at the doses that were effective in altering cocaine self-administration measures.

## Discussion

In contrast to previous findings demonstrating a leftward shift in the cocaine self-administration dose-effect function with selective 5-HT_1B_R agonists given during daily maintenance sessions^8,14^, the present findings show that the nonselective 5-HT_1D/1B_R agonist zolmitriptan decreased cocaine intake during maintenance sessions in both male and female rats when either a low (0.075 mg/kg, i.v.) or a high (0.75 mg/kg, i.v.) cocaine dose was available. Because the 0.075 mg/kg dose of cocaine is on the ascending limb of the cocaine dose-effect function^14^, an agonist-induced increase in intake at this dose is consistent with a leftward shift in the dose-effect function, whereas a decrease at this dose is consistent with a downward shift in the dose-effect function^32^. Thus, the zolmitriptan-induced decrease in intake at the low cocaine dose, together with the decrease at the high cocaine dose, is most likely a downward shift in the cocaine self-administration dose-effect function. This downward shift suggests that zolmitriptan inhibits cocaine reinforcement. Similarly, unpublished data from our laboratory in drug-experienced male rats (*n* = 9–13) found that zolmitriptan (10 mg/kg, s.c.) significantly decreased cocaine intake after a 21-day period of abstinence, where mean infusions (±SEM) obtained for 0.075 and 0.75 mg/kg, i.v., respectively after vehicle pretreatment were 84.00 (±9.65) and 17.63 (±1.08) versus 57.64 (±7.71) and 12.50 (±1.30) after zolmitriptan treatment. Collectively, the present and previous findings suggest that zolmitriptan inhibits cocaine reinforcement regardless of whether or not rats have undergone abstinence.

The effect of zolmitriptan on cocaine self-administration when the low cocaine dose was available showed an inverted U-shaped dose-response function, with decreases observed at the two intermediate doses and no effect observed at the highest dose of zolmitriptan. Inverted U-shaped dose-effect functions are not uncommon. Although the mechanism underlying the inverted U-shaped function in the present study is unclear, it may involve differences in extracellular endogenous monoamines across the two cocaine doses resulting in differences in receptor trafficking, desensitization, or intracellular signaling.

The zolmitriptan-induced attenuation in cocaine intake is not likely due to disruptive effects on operant behavior and/or general reinforcement. Zolmitriptan doses effective at reducing cocaine intake had no effect on sucrose intake and the lack of a zolmitriptan effect on locomotion suggests that motor function was intact. Previous research has shown that 5-HT_1B_R agonists either increase^33^ or produce no change in locomotor activity^16,34^. It is possible that previous experience with cocaine self-administration, as in the present study, desensitizes 5-HT_1B_R agonist-induced increases in locomotion that are sometimes observed in rodents. Consistent with this idea, we previously observed no change in locomotion with the selective 5-HT_1B_R agonist, CP94253, in cocaine-experienced rats^16^ and other studies have found that 5-HT_1B_Rs are regulated by cocaine self-administration and subsequent abstinence^4,35,36^. Alternatively, it is possible that zolmitriptan may have less of an effect on locomotion compared to other 5-HT_1B_R agonists as others have failed to observe an effect of zolmitriptan (3.0–30 mg/kg, i.p.) on locomotion^37^, although other triptans increase locomotion in male and female rats^38,39^. The lack of zolmitriptan effects on locomotion and sucrose intake mitigate the possibility that zolmitriptan nonspecifically altered motor capability or other processes involved in operant behavior.

The discrepancy between the effects of zolmitriptan and other selective 5-HT_1B_R agonists on cocaine reinforcement during the active drug-taking phase remains unclear. Differences in cocaine intake may arise from differences in receptor binding affinity of each compound and how each receptor modulates cocaine reinforcement. Zolmitriptan, like other triptans, displays affinity for both 5-HT_1B_Rs and 5-HT_1D_Rs^21^, and our results suggest that both receptor subtypes play a role in the zolmitriptan-induced decrease in cocaine intake. Indeed, the 5-HT_1B_R antagonist SB224289 dose-dependently reversed this effect, which was evident at a lower SB224289 dose when the high cocaine dose was available compared to when the low cocaine dose was available. While the effects of the 5-HT_1D_R antagonist BRL15572 were less robust than those of the 5-HT_1B_R antagonist SB224289, BRL15572 attenuated zolmitriptan effects when the high cocaine dose was available, and increased variability when the low cocaine dose was available regardless of BRL15572 dose. Furthermore, neither antagonist given alone altered cocaine intake regardless of the cocaine dose available. Perhaps the higher affinity of zolmitriptan for 5-HT_1D_Rs over 5-HT_1B_Rs contributes to its opposing effects compared to more selective 5-HT_1B_R agonists on cocaine intake during maintenance of daily self-administration. In further support of a role of 5-HT_1D_Rs in psychostimulant effects, Shahidi and colleagues found that administration of the 5-HT_1D_R agonist PNU142633 blocks methamphetamine-primed reinstatement of conditioned place preference^40^.

The contribution of 5-HT_1B_Rs vs. 5-HT_1D_Rs to the inhibitory effects of zolmitriptan and other 5-HT_1B_R agonists on cocaine reinforcement remains equivocal for a few reasons. Both 5-HT_1B_Rs and 5-HT_1D_Rs function as auto- and heteroreceptors and negatively couple to adenylyl cyclase activity via G-proteins that function to inhibit neurotransmitter release^41–45^. 5-HT_1B_Rs and 5-HT_1D_Rs are expressed in many of the same brain regions^46–50^ and both receptor subtypes share more than 60% amino acid sequence homology^51^. 5-HT_1B_Rs are expressed at higher levels in rodent brains relative to human brains and 5-HT_1D_Rs are expressed at higher levels in human brains relative to rodent brains^49,52^, leading to the suggestion that these receptors are functional analogs^53–55^. There are also differences in 5-HT_1B_R regulation between rodents and humans. In rats, 5-HT_1B_Rs are upregulated in numerous brain regions following a 5-day withdrawal period from chronic cocaine exposure^35^ and have increased sensitivity to a 5-HT_1B_R agonist challenge following 14 days of abstinence from cocaine^4^. In cocaine-dependent patients, 5-HT_1B_R availability decreases throughout multiple brain regions following 6 days of cocaine withdrawal^56^. These discrepancies in 5-HT_1B_R regulation may arise from differences in history of cocaine experience, length of withdrawal, or species. Nonetheless, our findings that both 5-HT_1B_Rs and 5-HT_1D_Rs play a role in cocaine intake suggests zolmitriptan effects may translate to CUDs in humans despite species differences in the relative proportion of 5-HT_1B_Rs to 5-HT_1D_Rs.

Future research investigating effects of chronic zolmitriptan treatment is needed to further advance the candidacy of zolmitriptan as treatment of CUDs. Repetitive treatment with zolmitriptan may produce tolerance, which may require incremental increases in dose to maintain efficacy. Here we found that zolmitriptan is effective at reducing cocaine intake following intermittent testing in rats prior to undergoing a period of abstinence. Furthermore, we previously demonstrated that zolmitriptan continues to be effective at reducing drug intake following three intermittent treatments during resumption of methamphetamine self-administration after prolonged abstinence in male rats^30^. These preliminary findings are encouraging to hypothesizing that zolmitriptan will be efficacious as a chronic treatment for CUDs.

A concern regarding the use of zolmitriptan for CUDs is that concomitant treatment with triptans and 5-HT reuptake inhibitors may precipitate the 5-HT syndrome, which can be life-threatening^57^. Serotonin syndrome is characterized by excessive 5-HT availability in the central nervous system and consists of autonomic hyperactivity, neuromuscular abnormalities, and changes in mental status^58–60^. In 2006, the FDA issued a warning indicating that triptans co-administered with selective monoamine reuptake inhibitors could precipitate the 5-HT syndrome^61,62^. However, this warning is based on a limited number of case studies from ~20 years ago, while in the intervening time there are no reports suggesting that zolmitriptan treatment either alone or with selective monoamine reuptake inhibitors induces 5-HT syndrome^57,62,63^. Studies investigating the interaction between other triptans (e.g. sumatriptan) and serotonin reuptake-inhibiting antidepressants on 5-HT syndrome have also produced negative results^64,65^. Despite the FDA report, the concomitant use of triptans with 5-HT indirect agonists is widespread and without indication of serious health consequences^62,66^.

In conclusion, the exciting discovery that zolmitriptan attenuates cocaine intake in rats regardless of sex and without a period of abstinence suggests that this drug may be suitable for repurposing as a medication for CUDs and perhaps other psychostimulant use disorders. Both 5-HT_1B_Rs and 5-HT_1D_Rs may contribute to the efficacy of zolmitriptan in reducing cocaine intake and studies examining potential toxicity effects of zolmitriptan and cocaine in humans is warranted.

## Supplementary information

Supplementary Figure
