# Supplementary material for: Preclinical support for the therapeutic potential of zolmitriptan as a treatment for cocaine use disorders
Source: Transl Psychiatry. 2020 Aug 3;10:266. doi: 10.1038/s41398-020-00956-6 (PMC7398918; doi:10.1038/s41398-020-00956-6)
Supplement: Supplementary file 1 — Supplementary Figure [file 41398_2020_956_MOESM1_ESM.docx]

Supplementary Information

**Figure S1.** Effects of cocaine self-administration in male and female rats under testing conditions with the 5-HT1D receptor antagonist, BRL15572, and zolmitriptan. Mean (±SEM) for cocaine infusions **(A)** and active lever responses **(B)** during test sessions where the training dose of cocaine (0.75 mg/kg, i.v.) was available for 1 h followed by a low dose of cocaine (0.075 mg/kg, i.v.) available for 1 h. Rats (n = 18-28/dose) were pretreated 30 min prior to the start of the test sessions with BRL15572 (0.3-3 mg/kg, i.p.) followed by either vehicle or zolmitriptan (5.6 mg/kg, s.c.)15 min later. Statistical analyses revealed a cocaine dose by sex interaction, asterisks (*) represent a difference from male rats (p < 0.05).
